# Assessment of health risks from exposure to indoor volatile organic compounds in European educational buildings

**DOI:** 10.1038/s41598-026-37072-2

**Published:** 2026-01-28

**Authors:** Anoushka Chatterjee, László Pál, Szabolcs Lovas, Martin McKee, Judit Diószegi, Nóra Kovács, Sándor Szűcs

**Affiliations:** 1 Department of Public Health and Epidemiology, Faculty of Medicine, University of Debrecen, P.O. Box 9, Debrecen, H–4012 Hungary; 2 Department of Health Services Research and Policy, London School of Hygiene and Tropical Medicine, London, UK

**Keywords:** Indoor air quality, Educational buildings, Risk assessment, Benzene, Formaldehyde, Diseases, Environmental sciences, Health care, Health occupations, Risk factors

## Abstract

Volatile organic compounds (VOCs) comprise an important group of indoor air pollutants, commonly found in building materials and consumer products. Due to their low boiling points, VOCs are prevalent in indoor environments, with concentrations in homes, schools, and offices often two to five times higher than outdoors. Chronic exposure to VOCs is linked to a range of adverse health outcomes, including respiratory, neurological, cardiovascular damage, and an increased cancer risk. Children and adolescents who spend a significant amount of time in educational buildings are particularly vulnerable to these effects. Therefore, this study aimed to estimate the related health risks to those in day care centres, schools, high-schools and universities across 17 member states of the European Union (EU) by utilizing a previously published dataset and collecting data on levels of 9 VOCs. Health risks were assessed using the World Health Organization’s Indoor Air Quality Risk Calculator. Point of departure indices (PODIs) and cancer risks were calculated, with values above 1.0 and 10 cases/1 million population respectively indicating an increased health risk. Our results showed that formaldehyde exposure poses an increased risk of respiratory, neurological and carcinogenic effects in educational buildings in 14 of the countries studied. Additionally, neurological risks from exposure to benzene were above the limit in 4 EU countries. These findings indicate the need to reduce formaldehyde and benzene concentrations in European educational buildings to protect the health of the next generation.

**Supplementary Information:**

The online version contains supplementary material available at 10.1038/s41598-026-37072-2.

## Introduction

Indoor air pollution has been identified as a significant public health problem worldwide, with a non-negligible contribution to the overall burden of disease^1,2^. Survey data indicate that people spend approximately 90% of their time indoors, although where they spend their time, in homes, offices, schools, and other public and private buildings varying by age, employment, and other characteristics^3,4^. Globally, the World Health Organization (WHO) estimates that 3.2 million people die prematurely each year from diseases related to exposure to a range of indoor air pollutants^5^. To inform measures to reduce these risks, the WHO has established guideline values for indoor air quality (IAQ) for individual chemical air pollutants^6,7^. Most of the substances listed in the guideline are organic compounds, an epidemiologically important class of hazardous indoor air pollutants that can cause both acute and chronic health effects^1,6–8^. The WHO classifies these chemicals as very volatile organic compounds, volatile organic compounds (VOCs) and semi-volatile organic compounds (SVOCs) characterised by boiling points ranging from below 0 °C to 50–100 °C, from 50–100 °C to 240–260 °C, and from 240–260 °C to 380–400 °C, respectively^9,10^. Aldehydes (formaldehyde, acetaldehyde), aromatic hydrocarbons (benzene, ethylbenzene, toluene, xylene), chlorinated hydrocarbons (trichloroethylene, tetrachloroethylene) and esters (n-butyl acetate) are among the VOCs most frequently detected indoors^6,7^. Their low boiling point and ubiquitous presence, in many industrial and consumer products including building materials, furniture, carpets, dyes, air fresheners, paint solvents, adhesives, household cleaning products, cosmetics, and electronic equipment^11–13^, give rise to concentrations in homes, schools, and offices that are 2 to 5 times higher than outdoors^4,14,15^. In addition, some of the VOCs found indoors come from outside, especially from road traffic emissions^13–15^. This wide variety of sources and differences in building characteristics mean that people are often exposed indoors to a complex mixture of harmful VOCs^16–18^.

The problem has been exacerbated in recent years as efforts to improve energy efficiency have led to buildings becoming increasingly sealed from the external environment, thereby reducing heating and cooling costs^19,20^. As a result, many buildings now rely solely on mechanical ventilation systems that recirculate indoor air with minimal addition exchange of fresh air^19,20^, thereby contributing to their accumulation.

Acute exposure to VOCs can result in several adverse effects, including irritation of the eyes, nose, and throat, due to their interaction with mucous membranes and sensory receptors^21,22^. They can also react with mucin glycoproteins, causing IgE-mediated inflammation and epithelial damage to the respiratory tract^23^. This can lead to painful breathing and can exacerbate asthma, and cause damage to the cardiovascular and central nervous systems^23,24^. Associated symptoms include headache, dizziness, and fatigue^23,25–28^.

Long-term exposure to VOCs has been shown to damage the respiratory system resulting in chronic bronchitis, reduced lung function, and progression of asthma^11,21,25,29^. As VOCs are lipid soluble, they cross the blood–brain barrier^23,30^ and can influence neurotransmitter functions and induce oxidative stress in neural tissues. In these ways, chronic VOC exposure can impair cognitive functions, memory, and peripheral nerve signalling^21,23,30^. Metabolites of VOCs have been reported to cause cardiovascular injury^24,27^, promoting endothelial dysfunction, hypertension, and atherosclerosis, and increasing the risk of cardiovascular diseases^24,27^. Chronic exposure to specific VOCs has also been identified as a risk factor for various cancers^21–23^. For example, benzene, formaldehyde and trichloroethylene have been classified by the International Agency for Research on Cancer (IARC) as carcinogenic to humans (Group 1), associated with increased incidence of myeloid leukaemia, nasopharyngeal and kidney cancer, respectively^31–33^.

Children and adolescents are particularly vulnerable to the health hazards associated with chronic exposure to VOCs^34,35^. Their respiratory and immune systems are still developing, making them less able to metabolise and eliminate toxic substances^34,35^. In addition, they have a higher respiratory rate relative to their bodyweight, which can lead to increased inhalation of air pollutants^34,35^. The amount of time they spend in educational buildings, typically 6 hours a day, 5 days a week for many months of the year, places them at increased risk from indoor air pollutants such as VOCs, potentially leading to respiratory problems, allergic diseases, and long-term developmental complications^34–36^. Yet despite this growing body of evidence on health risks associated with both short- and long-term exposure these pollutants^21,22^, exposure indoors has attracted less attention than their presence in the ambient air^21^.

The potential risks are especially great in educational facilities. Like any workplace, day care centre, elementary school, high-school and university buildings contain VOC-emitting items, such as carpets and furniture. However, they have an increased likelihood of having products like adhesives, paints, and cleaning agents, all important sources of VOCs^37^. Inadequate ventilation in classrooms can exacerbate this problem, allowing pollutants to accumulate over time^37^. For these reasons, this study investigates and quantifies the health risk in these settings by collecting data on VOC exposure levels in European day care centres, elementary schools, high-schools and university buildings. To our knowledge, this is the first study that used the Indoor Air Quality (IAQ) Risk Calculator, a screening tool developed by the WHO Regional Office for Europe and the European Centre for Environment and Health, to estimate the health risks associated with exposure to indoor air pollutants.

## Data and methods

We extracted data on VOC levels in educational buildings in European Union (EU) member states from our previously published dataset on VOC concentrations in indoor environments of offices, educational buildings and residential buildings in the EU between 2010 and 2023^38^. Records with data on levels of VOCs in day-care centres, kindergartens, elementary and high schools, and universities were selected to prepare a separate database for health risk assessment.

### Development of a database for health risk assessment

Data were extracted from 28 articles and used to generate a comprehensive database of VOC concentrations in educational buildings. The database included information on the authors, the titles of the articles, the concentrations of VOCs, and the type and number of buildings for countries in the EU. Our database contained data from 17 European countries: Croatia, Cyprus, the Czech Republic, Finland, France, Germany, Greece, Hungary, Ireland, Italy, the Netherlands, Poland, Portugal, Romania, Slovenia, Spain, and Sweden. The 9 VOCs, included formaldehyde, acetaldehyde, benzene, ethylbenzene, o, m-, and p-xylenes, styrene, toluene, 1,4-dichlorobenzene, and trichloroethylene. When data on the level of VOCs was reported from multiple buildings within the same study, the mean value was calculated and entered into the database.

### Health risk assessment

The IAQ Risk Calculator software (Indoor Air Quality Risk Calculator, version 1.0.0.0; https://www.who.int/europe/tools-and-toolkits/indoor-air-quality-(iaq)-riskcalculator--assessing-risks-for-children-s-health-from-chemical-indoor-air-pollution) was used to estimate the health risks associated with indoor exposure to the selected VOCs^39^. This is a user-friendly tool that assesses health risks from combined exposure to indoor air pollutants in public places, such as schools, for children^39^. To achieve this, the tool incorporates tiers modified from those of the WHO framework for assessing exposure to multiple chemicals^39^. It includes risk calculation spreadsheets and a supporting database of guideline values containing information on points of departure (PODs) for inhalation for selected effects^39,40^. The terminology and approach of the screening tool are consistent with the International Programme on Chemical Safety (IPCS) and the WHO IPCS framework^41,42^. In assessing hazards, the tool uses modified early stages (stages 0 and 1) of the WHO IPCS framework^41^. The concentrations of various individual VOCs in EU countries, as reported in the database, were entered into the IAQ Risk Calculator, yielding article-specific results. The substances included in the supporting toxicological database for the screening tool are those for which co-exposure in indoor air in public settings is most likely, particularly among children^39,40^. The PODs included in this database involve the respiratory, nervous and cardiovascular systems, as well as carcinogenicity of IARC group 1 carcinogens^39,40^.

The WHO IPCS risk assessment approach is based on a tiered assessment strategy^39,40^. Assessments start with relatively simple and often conservative evaluations in the early tiers and progress to more complex and refined estimations in subsequent tiers as deemed necessary^39,40^. This hierarchical system is designed to guide risk assessors through a systematic evaluation process^39,40^. Each tier represents an increasing level of complexity and refinement in both exposure and hazard assessment^39,40^. Tier 0 is the initial screening stage^39,40^. At this point, the assessment is typically based on limited information about the specific chemicals under consideration^39,40^. Exposure estimates in Tier 0 are primarily derived from the fundamental physicochemical properties of the substances and their potential exposure routes^39,40^. This provides an initial, rapid filter to identify substances that could plausibly give rise to concern^39,40^. At this stage, the software calculates a hazard index (HI)^39,40^, defined as the sum of the hazard quotients (HQs), i.e., the level of exposure (the concentration of the substance in question) to each of the constituents in an assessment group (AG) divided by its respective reference concentration (RC). An AG is a set of substances that are evaluated together because they might affect the same organ or system in the body. The RC is the maximum safe level of that substance, based on the most sensitive health effect it can cause^39,40^. HI can be calculated using the following equation:

$$HI = \sum\limits_{{x = 1}}^{n} {\frac{{{\mathrm{measured}}\;{\mathrm{concentration}}_{x} }}{{RC^{x} }}}$$

where.

*x* = each substance included in the AG, irrespective of its health effects,

RC = reference concentration for inhalation based on critical effect.

To calculate HI, we used the most recent reference concentrations from the WHO and the Agency for Toxic Substances and Disease Registry^39,40^. A HI greater than 1 requires a refined risk assessment or consideration of corrective measures to reduce exposure^39,40^. Tier 1, Level 1 is applied to those exposures that pass the Tier 0 screening and involves a more detailed assessment of exposure and hazard^39,40^. At this stage, the evaluation considers information on chemicals that goes beyond their basic physicochemical properties^39,40^. Tier 1 exposure estimates are usually derived from exposure modelling results^39,40^. This information may include details on the exposed population, exposure routes, the environmental fate of the substances, production volumes, and average values from air quality monitoring databases^39,40^. In this case, the software calculates the hazard index assessment group (HI_ag_) in the same way as for Tier 0 but categorises chemicals based on five selected adverse effects of indoor air pollution, including respiratory, cardiovascular, neurological, irritative, and carcinogenic effects^39,40^.

HI_ag_ can be calculated according to the following equation:

$$HI_{{ag}} = \sum\limits_{{x = 1}}^{n} {\frac{{{\mathrm{measured}}\;{\mathrm{concentration}}_{x} }}{{RC^{x} }}}$$

where.

*ag* = substances grouped for evaluation for one of the designated priority health effects,

*x* = each substance included in the assessment group,

RC = reference concentration for inhalation for the relevant substance, for example, WHO guidance values.

The HI_ag_ was calculated using the same reference concentrations as in Tier 0. At Tier 2, the software calculates an adjusted point of departure index (PODI_adj_) for the selected effects of interest, which include respiratory, cardiovascular, neurological, and irritative effects^39,40^. The PODI_adj_ is based on the lowest point of departure (POD)^39,40^. The POD is defined as the dose or concentration chosen as the point of comparison for exposure estimates and serves as a basis for risk assessment^39,40^. The POD can be the “no observed adverse effect level” (NOAEL), the “lowest observed adverse effect level” (LOAEL), or the benchmark dose or benchmark concentration^39,40^. When data are available from both human studies and animal experiments, the lowest dose or concentration is used to calculate the PODI_adj_^39,40^. If data are only available from animal studies, the POD values are divided by the conventional default uncertainty factor (UF) of 10 to account for possible differences between humans and other species^39,40^. The NOAEL is used in calculations wherever possible^39,40^. If only the LOAEL is available, the screening tool divides this value by three^39,40^. Human variability is accounted for by dividing the POD by ten^39,40^. For systemic effects, PODs determined for chronic exposure are preferred^39,40^. If these values are unavailable, the shorter exposure duration is considered by adjusting the POD values by a factor of two^39,40^.

PODI_adj_ can be calculated using the following equation:

$$PODI_{{adj}} = \sum\limits_{{x = 1}}^{n} {\frac{{{\mathrm{measured}}\;{\mathrm{concentration}}_{x} }}{{PODxadj}}}$$

where.

*PODI*_*adj*_ = the PODI adjusted for an assessment group for each of the priority effects,

*PODx adjusted* = the POD for the relevant priority health effect for substance x adjusted by period of exposure (e.g., intermediate vs. chronic) and an acceptable margin to account for uncertainty.

If the PODI_adj_ is greater than 1, the assessment should be refined or corrective measures should be considered to reduce exposure^39,40^. In our risk assessment, the PODI_adj_ was calculated using the lowest POD value obtained from epidemiological or animal studies^39,40^.

Some of the chemicals in the IAQ database are classified as IARC Group 1 carcinogens^39,40^. For these compounds, the software also calculates the PODI_adjcancer_ value based on the tumorigenic concentration of 50 (TC50), which is the concentration associated with a 50% increase in cancer risk^39,40^. If the TC50 values are from animal experiments or human studies, they are divided by 50,000 or 5,000, respectively^39,40^. This gives a cancer risk of 10 in 10^6^ (ten cancer case per 1 million population). These values align with the limits established by the European Food Safety Authority as low-priority for the risk management of carcinogenic and genotoxic substances^43^.

PODI_adjcancer_ can be calculated using the following equation:

$$PODI_{{adjcancer}} = \sum\limits_{{x = 1}}^{n} {\frac{{{\mathrm{measured}}\;{\mathrm{concentration}}_{x} }}{{TC50xadj}}}$$

where.

*PODI*_*adjcancer*_ = the PODI adjusted for an assessment group for cancer,

*TC50x* = the concentration associated with a 50% increase in cancer risk, adjusted by an acceptable margin.

If the PODI_adjcancer_ is more than 10 cases per 1 million population, the assessment should be refined or remedial measures should be taken to reduce exposure^39,40^. Using the separate database prepared from our previously published dataset, the VOC concentrations measured in EU countries were entered into the IAQ Risk Calculator and used to calculate the PODI values for irritation, as well as for respiratory, neurological, cardiovascular and carcinogenic effects^39,40^. The results are presented in Tables 1 and 2.

**Table 1:** PODI_adj_ values for respiratory, neurological, and irritation effects due to indoor benzene exposure in educational buildings in the member states of European Union.

benzene exposure						
author	country	number of buildings studied	mean concentration [mg/m^3^]	PODI_adj_ respiratory effects	PODI_adj_ neurological effects	PODI_adj_ irritation effects
Brdarić et al.^44^	Croatia	2	0.00104	0.0003	0.2506	0.0003
Geiss et al.^45^	Cyprus	3	0.00307	0.0009	0.7398	0.0009
Szabados et al.^46^	Czechia	12	0.00347	0.0010	0.8361	0.0010
Geiss et al.^45^	Finland	3	0.00115	0.0003	0.2771	0.0003
Canha et al.^47^	France	17	0.00210	0.0006	0.5060	0.0006
Ramalho et al.^48^	France	310	0.00232	0.0007	0.5590	0.0007
Verriele et al.^49^	France	10	0.00130	0.0004	0.3133	0.0004
Geiss et al.^45^	Germany	3	0.00622	0.0018	**1.4988**	0.0018
Geiss et al.^45^	Greece	6	0.00533	0.0015	**1.2843**	0.0015
Geiss et al.^45^	Hungary	6	0.00573	0.0016	**1.3807**	0.0016
Szabados et al.^46^	Hungary	15	0.00461	0.0013	**1.1108**	0.0013
Geiss et al.^45^	Ireland	2	0.00358	0.0010	0.8627	0.0010
Geiss et al.^45^	Italy	3	0.00320	0.0009	0.7711	0.0009
Ielpo et al.^50^	Italy	1	0.00194	0.0006	0.4675	0.0006
Lucialli et al.^51^	Italy	8	0.00153	0.0004	0.3687	0.0004
Marzocca et al.et al.^52^	Italy	1	0.00043	0.0001	0.1036	0.0001
Romagnoli et al.^53^	Italy	1	0.00092	0.0003	0.2217	0.0003
Szabados et al^46^	Italy	11	0.01090	0.0031	**2.6265**	0.0031
Geiss et al.^45^	Netherlands	2	0.00170	0.0005	0.4096	0.0005
Mainka et al.^54^	Poland	2	0.00232	0.0007	0.5590	0.0007
Szabados et al.^46^	Poland	11	0.00306	0.0009	0.7373	0.0009
Pegas et al.^55^	Portugal	14	0.00069	0.0002	0.1663	0.0002
Fonseca et al.^56^	Portugal	20	0.00100	0.0003	0.2410	0.0003
Pegas et al.^57^	Portugal	1	0.00031	0.0001	0.0747	0.0001
Szabados et al^46^	Slovenia	12	0.00415	0.0012	1.0000	0.0012
Lizana et al.^58^	Spain	6	0.00123	0.0004	0.2964	0.0004
Ninyá et al.^59^	Spain	1	0.00048	0.0001	0.1157	0.0001
Vallecillos et al.^60^	Spain	1	0.00041	0.0001	0.0988	0.0001
Villanueva et al.^61^	Spain	18	0.00053	0.0002	0.1277	0.0002

. PODI_adj_ values greater than 1 are shown in bold and indicate studies where there was an increased health risk.

**Table 2:** PODI_adj_ values for respiratory, cardiovascular, neurological, and carcinogenic effects due to indoor formaldehyde exposure in educational buildings in the member states of European Union.

formaldehyde exposure							
author	country	number of buildings studied	mean concentration [mg/m^3^]	PODI_adj_ respiratory effects	PODI_adj_ cardiovascular effects	PODI_adj_ neurological effects	cancer cases/1 million population
Brdarić et al.^44^	Croatia	2	0.00848	0.8653	0.0482	0.1381	10
Geiss et al.^45^	Cyprus	3	0.01200	**1.2245**	0.0682	0.1954	**20**
Szabados et al.^46^	Czechia	12	0.00779	0.7949	0.0443	0.1269	10
Geiss et al.^45^	Finland	3	0.01040	**1.0612**	0.0591	0.1694	**20**
Hu et al.^63^	France	3	0.01372	**1.4000**	0.078	0.2235	**20**
Ramalho et al.^48^	France	310	0.01873	**1.9112**	0.1064	0.3050	**30**
Geiss et al.^45^	Germany	3	0.03080	**3.1429**	0.175	0.5016	**50**
Geiss et al.^45^	Greece	6	0.01731	**1.7663**	0.0984	0.2819	**30**
Geiss et al.^45^	Hungary	6	0.01520	**1.5510**	0.0864	0.2476	**20**
Szabados et al.^46^	Hungary	16	0.00867	0.8847	0.0493	0.1412	10
Geiss et al.^45^	Ireland	2	0.01024	**1.0449**	0.0582	0.1668	**20**
Szabados et al^46^	Italy	12	0.00980	1.0000	0.0557	0.1596	**20**
Geiss et al.^45^	Italy	3	0.01430	**1.4592**	0.0812	0.2329	**20**
Geiss et al.^45^	Netherlands	2	0.01393	**1.4214**	0.0791	0.2269	**20**
Szabados et al.^46^	Poland	12	0.00773	0.7888	0.0439	0.1259	10
Branco et al.^64^	Portugal	4	0.01640	**1.6735**	0.0932	0.2671	**30**
Branco et al.^65^	Portugal	25	0.01340	**1.3673**	0.0761	0.2182	**20**
Ferreira et al.^66^	Portugal	51	0.01837	**1.8745**	0.1044	0.2992	**30**
Fonseca et al.^56^	Portugal	20	0.01690	**1.7245**	0.096	0.2752	**30**
Nunes et al.^67^	Portugal	4	0.05157	**5.2622**	0.293	0.8399	**80**
Oliviera et al.^68^	Portugal	2	0.03500	**3.5714**	0.1989	0.5700	**60**
Sá et al.^62^	Portugal	5	0.07167	**7.3133**	0.4072	**1.1673**	**110**
Neamtiu et al.^69^	Romania	5	0.03416	**3.4857**	0.1941	0.5564	**50**
Szabados et al.^46^	Slovenia	12	0.01150	**1.1735**	0.0653	0.1873	**20**
Ninyá et al.^59^	Spain	1	0.00883	0.9010	0.0502	0.1438	10
Villanueva et al.^61^	Spain	18	0.02699	**2.7541**	0.1534	0.4396	**40**
Cabovská et al.^70^	Sweden	23	0.01083	**1.1051**	0.0615	0.1764	**20**
Wang et al.^71^	Sweden	39	0.00725	0.7398	0.0412	0.1181	10

More than 10 cancer cases/1 million population and PODI_adj_ values greater than 1 are shown in bold and indicate studies where there was an increased health risk.

## Results

For 7 of the 9 VOCs examined, the PODI_adj_ values for respiratory, cardiovascular, neurological, and carcinogenic effects were less than one (see data in Supplement 1). The remaining two were formaldehyde and benzene and, in their cases, the calculated PODI_adj_ values for respiratory and neurological effects exceeded one in several cases. Furthermore, in multiple instances, the cancer risk associated with exposure to formaldehyde equalled or exceeded 10 cases per 1 million population. As exposure to formaldehyde and benzene posed the highest health risk, the results obtained for these VOCs are presented in Tables 1 and 2.

### Health risks attributable to indoor benzene exposure

The PODI_adj_ values for respiratory, neurological, and irritation effects due to indoor exposure to benzene are presented in Table 1. As can be seen, the PODI_adj_ values for respiratory and irritation effects were low, ranging from 0.0001 to 0.0031 in both categories. In addition, the PODI_adj_ values for neurological effects from benzene exposure varied between 0.0747 and 2.6265, with values greater than 1 indicating an increased health risk in 3, 6, 21 and 11 school buildings in Germany, Greece, Hungary, and Italy, respectively. In all the studies included in our research, the cancer risk from exposure to benzene in educational buildings was below the maximum acceptable level of 10 cases of cancer per 1 million population.

### Health risks attributable to indoor formaldehyde exposure

The PODI_adj_ values for respiratory, neurological, cardiovascular, and carcinogenic effects resulting from indoor formaldehyde exposure are presented in Table 2. As shown, the PODI_adj_ values for respiratory effects ranged from 0.7398 to 7.3133. An increased risk of respiratory effects due to formaldehyde exposure was found in a varying number of educational buildings when analysing data obtained in Cyprus (n = 3), Finland (n = 3), France (n = 313), Germany (n = 3), Greece (n = 6), Hungary (n = 6), Ireland (n = 2), Italy (n = 3), the Netherlands (n = 2), Portugal (n = 111), Romania (n = 5), Slovenia (n = 12), Spain (n = 18), and Sweden (n = 23). Additionally, based on the concentration data collected in 5 school buildings in Portugal by Sá et al., 2019, the PODI_adj_ values for neurological effects due to formaldehyde exposure were greater than one^62^. The cancer risk from formaldehyde exposure ranged from 10 to 110, exceeding the maximum acceptable level of 10 cancer cases per 1 million population in educational buildings in Cyprus (n = 3), Finland (n = 3), France (n = 313), Germany (n = 3), Greece (n = 6), Hungary (n = 6), Ireland (n = 2), Italy (n = 15), the Netherlands (n = 2), Portugal (n = 111), Romania (n = 5), Slovenia (n = 12), Spain (n = 18), and Sweden (n = 23).

## Discussion

This study reveals a concerning pattern of indoor air pollution in educational buildings in 17 EU member states, with formaldehyde and benzene emerging as the most significant threats to children’s health^35,37^. Using the WHO IAQ Risk Calculator, we found that formaldehyde levels in schools, kindergartens, and universities exceeded thresholds for respiratory and carcinogenic effects in 14 countries. Benzene exposure, while below cancer risk thresholds, posed notable neurological risks in four countries. These findings are particularly troubling given the vulnerability of children and adolescents, who spend a substantial portion of their day during term times in these environments^3,35^. Their developing respiratory and immune systems, combined with higher inhalation rates, make them more susceptible to the harmful effects of VOCs. Taken together, this evidence points to an urgent need for a comprehensive response to protect the health of future generations^72–74^.

The first step is to raise awareness of the sources of each substance. Benzene comes from indoor and outdoor sources, with significant spatial and seasonal variations^6,51,75^. The main indoor sources are building materials and furnishings, including varnishes, paints, adhesives, and urea–formaldehyde resins found in pressed wood products^6,51,75^. Floor covers have also been identified as major contributors to indoor benzene pollution^6^. A multinational study performed in Central Europe has found 2.1 times higher benzene concentrations in carpet covered Italian classrooms than in schools in other countries with alternative floor covers^46^. Therefore, it can be reasonable to assume that the elevated benzene levels were partially due to its release from synthetic carpets. Cleaning products and solvents can also contribute, so that levels may be higher in buildings that have recently undergone renovations or deep cleans^6,51,75^. Consumer products such as air fresheners, glues, and personal care items can release benzene either directly or through chemical reactions with indoor air components, such as ozone^6,51,75^. Urban schools, especially if situated near high-volume traffic or industrial sites are at additional risk from vehicle exhaust and industrial emissions^6,49,73^. The relative importance of these sources can be seen from studies of the ratio of outdoor to indoor benzene concentrations, which is often less than 1.0^76^. Seasonal factors can exacerbate exposure, as winter months bring higher levels due to reduced ventilation and prolonged classroom occupancy^9,77^. Benzene concentrations can be further increased by emissions from car parks, garages or idling vehicles near schools^6,75,77^.

Formaldehyde comes mainly from building materials and furnishings^6,75,78^, with concentrations influenced by the age of the building, ventilation efficiency, and environmental factors^6,75,78^. Primary sources include pressed wood products, particleboard, and plywood made with urea–formaldehyde resins, as well as carpets and flooring, which release formaldehyde vapour, especially when they are new^6,75,78^.

Other significant sources include cleaning products containing terpenes that can react with ozone in the outdoor air to produce formaldehyde, as well as residual tobacco smoke contamination that increases formaldehyde levels in classrooms and car exhaust fumes entering school buildings located next to busy roads^6,32,75,78^. This may provide a potential explanation for the findings of Sá et al. (2019), which demonstrated that formaldehyde levels in schools in the urban area of Porto exceeded threshold values^62^. This indicates that outdoor air pollution may have a substantial impact on indoor formaldehyde levels. As with benzene, there are also seasonal variations, with higher concentrations in classrooms during the summer months^6,51,75,78^. An optimal response to poor indoor air quality has four elements. The first comprises regulatory measures. These include enforcement of compliance with WHO indoor air quality guidelines^74^, mandating pre-occupancy air quality testing in new or renovated educational buildings^6,71,73,76^, and introduction of VOC emission limits for building materials and furnishings used in schools. For example, institutional policies should also restrict the use of cleaning products containing formaldehyde precursors and strictly enforce smoke-free policies^6,73,76^ and replace benzene-containing products^70,71^. The second element includes measures related to infrastructure and design. Here, a first step is to eliminate or reduce the number of sources, by removing building materials emitting VOCs or requiring constructors to the use of low-emitting alternatives^80^. The next step involves the development of a comprehensive indoor air quality management plan (IAQMP) to prevent, identify and resolve indoor air quality problems in school buildings^80^. Although improving ventilation has been shown to reduce VOC levels in school environments, this can only be effective if it is integrated into the IAQMP^70,71,76,80^. Mechanical ventilation with heating, ventilation, and air conditioning (HVAC) systems can significantly reduce indoor benzene and formaldehyde concentrations^70,71,76,80^. These measures have the significant added benefit of reducing exposure to airborne respiratory viruses. Activated carbon filtration in air purification devices is also effective^79^. Where high levels of formaldehyde persist, specific abatement technologies can be used, such as formaldehyde-catalysed activated carbon filtration systems^79^. However, they should not be considered as a general method for solving indoor air quality problems due to their significant limitations^82^. For example, filtration systems are often pollutant-specific and cannot address many gaseous compounds or persistent SVOCs at the same time^82^. The effectiveness of such measures can be monitored using formaldehyde-specific detectors, which will be especially useful in areas with limited air circulation^6,75,78^. The third element involves education and awareness raising. Regulations will only work if people understand why they have been adopted so specific and targeted training of educate school staff and students about sources of benzene, emphasising the risks associated with indoor smoking (which should never happen in an educational establishment anyway) and the improper storage of solvents and other chemicals containing benzene^72,73^. The final element is monitoring and evaluation. This calls for national or regional programmes for regular indoor air quality monitoring in educational facilities, coupled with public databases to track VOC levels and remediation efforts and research into long-term health effects and effectiveness of interventions to reduce exposures.

### Strengths and limitations

One strength of our study is that the database could be used to identify VOCs that pose a health risk to children and adolescents in EU member states. While it was not possible to quantify the health risks associated with non-cancer effects of VOCs, we were able to conduct a quantitative cancer risk assessment for formaldehyde and benzene. Another strength is that our estimation is based on data from the supporting toxicological database of the IAQ Risk Calculator, which was developed through extensive international collaboration^39^.

The limitations of our investigation must also be considered. Firstly, the number of educational buildings from which levels of VOCs were reported and used in our analysis has varied considerably by country. In addition, when multiple investigations were available from the same country, indoor air quality problems were often identified only in specific cases, indicating local problems with indoor VOC pollution. For example, the database contains seven studies from Portugal, representing 111 buildings. In six of the studies, comprising 106 of these buildings, the PODI_adj_ values for neurological effects due to formaldehyde exposure were less than 1. Only one study, including five buildings, had a value above 1. Consequently, the results of our analysis are only applicable to the children being in these buildings and they may not be generalised to the entire child population residing in the country where concentration data were collected. Secondly, our data were taken from research that determined the level of VOCs in day care centres, elementary schools, high-schools and universities using passive air sampling exclusively. Although passive sampling is the recommended approach for monitoring air quality in schools, it can only provide information on average VOC levels and is not suitable for measuring their peak concentrations^83^. Additionally, the IAQ Risk Calculator does not account for differences in inhalation rate or volume of air inhaled among age groups when calculating the PODI_adj_ values. Although the software developers considered these factors to have a negligible effect on the results, they may lead to an underestimation or overestimation of the health risks^39^. Similarly, no factor was introduced to convert intermittent exposure (five days per week during school term) to continuous exposure (all day, seven days per week), as the exposure patterns in the studies from which the reference concentrations and POD values were obtained closely resemble those experienced by schoolchildren (five days per week)^39^.

Assessing indoor air quality is a complex task. While health risk assessments are important, other factors that influence indoor pollutant levels must also be considered. One key factor is the contribution of outdoor (ambient) air pollution, which can be evaluated by monitoring outdoor air quality simultaneously with indoor measurements. This helps guide decisions about further monitoring, risk assessment, and mitigation strategies such as improving ventilation or controlling pollution sources. Given these complexities, risk assessment results should be interpreted with caution, and further research is recommended.

## Conclusions

This study highlights the substantial health risks associated with exposure to VOCs in European educational buildings. By systematically analysing reported VOC concentrations from 18 European countries and applying the WHO’s IAQ Risk Calculator, we identified several countries where formaldehyde and benzene exposure in schools emerged as major health concerns, particularly due to their potential respiratory and neurological effects. Given that children and adolescents spend a substantial portion of their day in educational settings, our findings highlight the need for targeted interventions to improve indoor air quality in these environments. This study adds to the growing body of evidence supporting the implementation of stricter regulations and proactive measures to reduce VOC exposure in schools. Future research should focus on the long-term health impacts of chronic, low-level VOC exposure and assess the effectiveness of mitigation strategies. Ensuring safe indoor air in educational buildings is not only a public health priority but also essential for protecting the well-being and development of future generations.

## Supplementary Information

Below is the link to the electronic supplementary material.

Supplementary Material 1

## Data Availability

The dataset used in this study are available on Mendeley Data: 10.17632/x4887snd8j.1.

## References

[1] Kumar, P., Singh, A. B., Arora, T., Singh, S. & Singh, R. Critical review on emerging health effects associated with the indoor air quality and its sustainable management. *Sci. Total Environ.* **872**, 162163 (2023). 36781134 10.1016/j.scitotenv.2023.162163

[2] Morantes, G., Jones, B., Molina, C. & Sherman, M. H. Harm from residential indoor air contaminants. *Environ. Sci. Technol.* **58**, 242–257 (2024). 38150532 10.1021/acs.est.3c07374 PMC10785761

[3] Schweizer, C. et al. Indoor time–microenvironment–activity patterns in seven regions of Europe. *J. Expo. Sci. Environ. Epidemiol.* **17**, 170–181 (2007). 16721413 10.1038/sj.jes.7500490

[4] Klepeis, N. E. et al. The National Human Activity Pattern Survey (NHAPS): A resource for assessing exposure to environmental pollutants. *J. Expo. Sci. Environ. Epidemiol.* **11**, 231–252 (2001). 10.1038/sj.jea.7500165 11477521

[5] World Health Organization, Household air pollution. (2024), https://www.who.int/news-room/fact-sheets/detail/household-air-pollution-and-health [accessed 12 December 2025]

[6] World Health Organization, WHO Guidelines for Indoor Air Quality: Selected Pollutants. (2010), World Health Organization, Regional Office for Europe, Copenhagen, Denmark 23741784

[7] World Health Organization, WHO Global Air Quality Guidelines: Particulate Matter (PM2.5 and PM10), Ozone, Nitrogen Dioxide, Sulfur Dioxide and Carbon Monoxide. (2021), World Health Organization, Geneva, Switzerland 34662007

[8] Settimo, G., Yu, Y., Gola, M., Buffoli, M. & Capolongo, S. Challenges in IAQ for indoor spaces: A comparison of the reference guideline values of indoor air pollutants from the governments and international institutions. *Atmosphere* **14**, 633 (2023).

[9] World Health Organization, Indoor Air Quality: Organic Pollutants Report on a WHO Meeting, Berlin (West), 23-27 August 1987. (1989), World Health Organization, Copenhagen, Denmark

[10] European Commission, European Collaborative Action ‘Indoor Air Quality and Its Impact on Man’: Total volatile organic compounds (TVOC) in Indoor air quality investigations. (1997), Publications Office, Luxembourg

[11] Chin, J.-Y. et al. Levels and sources of volatile organic compounds in homes of children with asthma. *Indoor Air* **24**, 403–415 (2014). 24329990 10.1111/ina.12086 PMC4057989

[12] Senthilnathan, J., Kim, K.-H., Kim, J.-C., Lee, J.-H. & Song, H. N. Indoor air pollution, sorbent selection, and analytical techniques for volatile organic compounds. *Asian J. Atmospheric Environ.* **12**, 289–310 (2018).

[13] You, B., Zhou, W., Li, J., Li, Z. & Sun, Y. A review of indoor gaseous organic compounds and human chemical exposure: Insights from Real-time measurements. *Environ. Int.* **170**, 107611 (2022). 36335895 10.1016/j.envint.2022.107611

[14] Adgate, J. L. et al. Outdoor, indoor, and personal exposure to VOCs in children. *Environ. Health Perspect.* **112**, 1386–1392 (2004). 15471730 10.1289/ehp.7107 PMC1247565

[15] Paciência, I., Madureira, J., Rufo, J., Moreira, A. & Fernandes, E. D. O. A systematic review of evidence and implications of spatial and seasonal variations of volatile organic compounds (VOC) in indoor human environments. *J. Toxicol. Environ. Health Part B* **19**, 47–64 (2016). 10.1080/10937404.2015.1134371 27163962

[16] Salthammer, T. & Bahadir, M. Occurrence, dynamics and reactions of organic pollutants in the indoor environment. *Clean: Soil, Air, Water* **37**, 417–435 (2009).

[17] Kotzias, D. & Sen, F. Official of the European Commission’s Joint Research Centre, Ispra/Italy, present address: Bonn/Germany Built environment and indoor air quality: The case of volatile organic compounds. *AIMS Environ. Sci.* **8**, 135–147 (2021).

[18] Horvat, T., Pehnec, G. & Jakovljević, I. Volatile Organic compounds in indoor air: Sampling, determination, sources, health risk, and regulatory insights. *Toxics* **13**, 344 (2025). 40423423 10.3390/toxics13050344 PMC12115474

[19] Yang, S. et al. Volatile organic compounds in 169 energy-efficient dwellings in Switzerland. *Indoor Air* **30**, 481–491 (2020). 32190933 10.1111/ina.12667 PMC7216845

[20] Szabados, M., Magyar, D., Tischner, Z. & Szigeti, T. Indoor air quality in Hungarian Passive Houses. *Atmos. Environ.* **307**, 119857 (2023).

[21] González-Martín, J., Kraakman, N. J. R., Pérez, C., Lebrero, R. & Muñoz, R. A state–of–the-art review on indoor air pollution and strategies for indoor air pollution control. *Chemosphere* **262**, 128376 (2021). 33182138 10.1016/j.chemosphere.2020.128376

[22] Halios, C. H. et al. Chemicals in European residences - Part I: A review of emissions, concentrations and health effects of volatile organic compounds (VOCs). *Sci. Total Environ.* **839**, 156201 (2022). 35623519 10.1016/j.scitotenv.2022.156201

[23] Ogbodo, J. O., Arazu, A. V., Iguh, T. C., Onwodi, N. J. & Ezike, T. C. Volatile organic compounds: A proinflammatory activator in autoimmune diseases. *Front. Immunol.* **13**, 928379 (2022). 35967306 10.3389/fimmu.2022.928379 PMC9373925

[24] Konkle, S. L. Volatile organic compound exposure and cardiometabolic syndrome risk in a nationally representative cohort. *Electronic Theses and Dissertations.* Paper 3409. (2020). 10.18297/etd/3409

[25] Lv, J. et al. Assessing volatile organic compounds exposure and chronic obstructive pulmonary diseases in US adults. *Front. Public Health* **11**, 1210136 (2023). 37475768 10.3389/fpubh.2023.1210136 PMC10354632

[26] Win-Shwe, T.-T., Fujimaki, H., Arashidani, K. & Kunugita, N. Indoor volatile organic compounds and chemical sensitivity reactions. *Clin. Dev. Immunol.* **2013**, 1–8 (2013). 10.1155/2013/623812 PMC3818819 24228055

[27] Han, S. et al. Associations between specific volatile organic chemical exposures and cardiovascular disease risks: insights from NHANES. *Front. Public Health* **12**, 1378444 (2024). 38846604 10.3389/fpubh.2024.1378444 PMC11153666

[28] Liu, N. et al. Health effects of exposure to indoor volatile organic compounds from 1980 to 2017: A systematic review and meta-analysis. *Indoor Air* **32**, e13038 (2022). 35622720 10.1111/ina.13038

[29] Paterson, C. A., Sharpe, R. A., Taylor, T. & Morrissey, K. Indoor PM2.5, VOCs and asthma outcomes: A systematic review in adults and their home environments. *Environ. Res.* **202**, 111631 (2021). 34224711 10.1016/j.envres.2021.111631

[30] Madaniyazi, L. et al. Early life exposure to indoor air pollutants and the risk of neurodevelopmental delays: The Japan Environment and Children’s Study. *Environ. Int.* **158**, 107004 (2022). 34991264 10.1016/j.envint.2021.107004

[31] International Agency for Research on Cancer Working Group on the Evaluation of Carcinogenic Risks to Humans. *Benzene*. (2020), International Agency for Research on Cancer, World Health Organization, Lyon, France

[32] International Agency for Research on Cancer Working Group on the Evaluation of Carcinogenic Risks to Humans. *Formaldehyde, 2-Butoxyethanol and 1-Tert-Butoxypropan-2-Ol.* (2006), International Agency for Research on Cancer, Lyon, France PMC4781641 17366697

[33] International Agency for Research on Cancer Working Group on the Evaluation of Carcinogenic Risks to Humans. *Trichloroethylene, Tetrachloroethylene, and Some Other Chlorinated Agents*. (2014), International Agency for Research on Cancer, Lyon, France PMC4781308 26214861

[34] World Health Organization. Regional Office for Europe & European Centre for Environment and Health. (2005), Effects of air pollution on children’s health and development: a review of the evidence. WHO Regional Office for Europe. Copenhagen, Denmark https://iris.who.int/handle/10665/107652 [last accessed 12 December 2025]

[35] United Nations Childrens Found. Breathless beginnings: the alarming impact of air pollution on children in Europe and Central Asia. (2023), UNICEF Europe and Central Asia Regional Office, Geneva, Switzerland

[36] Payus, C. M., Vasu Thevan, A. T. & Sentian, J. Impact of school traffic on outdoor carbon monoxide levels. *City Environ. Interact.* **4**, 100032 (2019).

[37] Sadrizadeh, S. et al. Indoor air quality and health in schools: A critical review for developing the roadmap for the future school environment. *J. Build. Eng.* **57**, 104908 (2022).

[38] Lovas, Sz. et al. Dataset on concentrations of volatile organic compounds in indoor environments of offices, educational and residential buildings in the European Union between 2010 and 2023. *Data Brief* **57**, 111070 (2024). 39583256 10.1016/j.dib.2024.111070 PMC11584602

[39] Meek, M. E., De Brouwere, K., Szigeti, T. & Zastenskaya, I. A user-friendly tool to assess combined exposures to indoor air pollutants in public spaces of children. *Food Chem. Toxicol.* **165**, 113141 (2022). 35588984 10.1016/j.fct.2022.113141

[40] World Health Organization. A Screening Tool for Assessment of Health Risks from Combined Exposure to Multiple Chemicals in Indoor Air in Public Settings for Children: Methodological Approach. (2021).

[41] Meek, M. E. et al. Risk assessment of combined exposure to multiple chemicals: A WHO/IPCS framework. *Regul Toxicol Pharmacol* **60**, S1–S14 (2011). 21466831 10.1016/j.yrtph.2011.03.010

[42] International Programme on Chemical Safety & Inter-Organization Programme for the Sound Management of Chemicals. Assessment of combined exposures to multiple chemicals : report of a WHO/IPCS international workshop on aggregate/cumulative risk assessment. 75 (2009).

[43] Opinion of the Scientific Committee on a request from EFSA related to A Harmonised Approach for Risk Assessment of Substances which are both Genotoxic and Carcinogenic. *EFSA J.*10.2903/j.efsa.2005.282.

[44] Brdarić, D. et al. Exposure assessment survey in schools: Pilot project in Osijek. *Croatia. J. Environ. Health* **82**, 14–21 (2020).

[45] Geiss, O. et al. The AIRMEX study - VOC measurements in public buildings and schools/kindergartens in eleven European cities: Statistical analysis of the data. *Atmos. Environ.* **45**, 3676–3684 (2011).

[46] Szabados, M. et al. Indoor air quality and the associated health risk in primary school buildings in Central Europe - The InAirQ study. *Indoor Air* **31**, 989–1003 (2021). 33615561 10.1111/ina.12802

[47] Canha, N., Lage, J., Coutinho, J. T., Alves, C. & Almeida, S. M. Comparison of indoor air quality during sleep in smokers and non-smokers’ bedrooms: A preliminary study. *Environ. Pollut. Barking Essex* **1987**, 248–256 (2019). 10.1016/j.envpol.2019.03.021 30893637

[48] Ramalho, O. et al. Association of carbon dioxide with indoor air pollutants and exceedance of health guideline values. *Build. Environ.* **93**, 115–124 (2015).

[49] Verriele, M. et al. The MERMAID study: indoor and outdoor average pollutant concentrations in 10 low-energy school buildings in France. *Indoor Air* **26**, 702–713 (2016). 26476191 10.1111/ina.12258

[50] Ielpo, P. et al. Air Quality Assessment of a School in an Industrialized Area of Southern Italy. *Appl. Sci.* **11**, 8870 (2021).

[51] Lucialli, P. et al. Indoor and outdoor concentrations of benzene, toluene, ethylbenzene and xylene in some Italian schools evaluation of areas with different air pollution. *Atmospheric Pollut. Res.* **11**, 1998–2010 (2020).

[52] Marzocca, A., Di Gilio, A., Farella, G., Giua, R. & de Gennaro, G. Indoor air quality assessment and study of different VOC contributions within a school in Taranto City, South of Italy. *Environ. * **4**, 1–11 (2017).

[53] Romagnoli, P. et al. Indoor air quality at life and work environments in Rome Italy.* Environ. Sci. Pollut. Res. Int.* **23**, 3503–3516 (2016). 26490929 10.1007/s11356-015-5558-4

[54] Mainka, A., Zajusz-Zubek, E., Kozielska, B. & Bragoszewska, E. Investigation of air pollutants in rural nursery school - A case study. *E3S Web Conf. ***28***, *01022 (2018).

[55] Pegas, P. N. et al. Seasonal evaluation of outdoor/indoor air quality in primary schools in Lisbon. *J. Environ. Monit.* **13**, 657 (2011). 21274462 10.1039/c0em00472c

[56] Fonseca Gabriel, M. et al. Environmental quality in primary schools and related health effects in children. An overview of assessments conducted in the Northern Portugal. *Energy Build.* **250**, 111305 (2021).

[57] Pegas, P. N. et al. Indoor and outdoor characterisation of organic and inorganic compounds in city centre and suburban elementary schools of Aveiro. *Portugal. Atmos. Environ.* **55**, 80–89 (2012).

[58] Lizana, J. et al. Contribution of indoor microenvironments to the daily inhaled dose of air pollutants in children. The importance of bedrooms. *Build. Environ.* **183**, 107188 (2020).

[59] Ninyà, N., Vallecillos, L., Marcé, R. M. & Borrull, F. Evaluation of air quality in indoor and outdoor environments: Impact of anti-COVID-19 measures. *Sci. Total Environ.* **836**, 155611 (2022). 35504390 10.1016/j.scitotenv.2022.155611 PMC9057935

[60] Vallecillos, L., Borrull, A., Marcé, R. M. & Borrull, F. Passive sampling to control air quality in schools: Uptake rate determination and application. *Indoor Air* **30**, 1005–1017 (2020). 32339338 10.1111/ina.12684

[61] Villanueva, F., Tapia, A., Lara, S. & Amo-Salas, M. Indoor and outdoor air concentrations of volatile organic compounds and NO_2_ in schools of urban, industrial and rural areas in Central-Southern Spain. *Sci. Total Environ.* **622–623**, 222–235 (2018). 29212055 10.1016/j.scitotenv.2017.11.274

[62] Sá, J. P., Branco, P. T. B. S., Alvim-Ferraz, M. C. M., Martins, F. G. & Sousa, S. I. V. Children’s exposure to indoor air in schools: Impact on wheezing. *WIT Trans. Ecol. Environ.* **236**, 205–212 (2019).

[63] Hu, D. et al. Diurnal variation and potential sources of indoor formaldehyde at elementary school, high school and university in the Centre Val de Loire region of France. *Sci. Total Environ.* **811**, 152271 (2022). 34902409 10.1016/j.scitotenv.2021.152271

[64] Branco, P. T. B. S., Nunes, R. A. O., Alvim-Ferraz, M. C. M., Martins, F. G. & Sousa, S. I. V. Children’s exposure to indoor air in urban nurseries – Part II: Gaseous pollutants’ assessment. *Environ. Res.* **142**, 662–670 (2015). 26342590 10.1016/j.envres.2015.08.026

[65] Branco, P. T. B. S., Alvim-Ferraz, M. C. M., Martins, F. G. & Sousa, S. I. V. Quantifying indoor air quality determinants in urban and rural nursery and primary schools. *Environ. Res.* **176**, 108534 (2019). 31220738 10.1016/j.envres.2019.108534

[66] Ferreira, A. M. C. & Cardoso, S. M. Estudo exploratorio da qualidade do ar em escolas de educacao basica, Coimbra. *Portugal. Rev. Saúde Pública* **47**, 1059–1068 (2013). 24626544 10.1590/s0034-8910.2013047004810 PMC4206112

[67] Nunes, R. A. O., Branco, P. T. B. S., Alvim-Ferraz, M. C. M., Martins, F. G. & Sousa, S. I. V. Gaseous pollutants on rural and urban nursery schools in Northern Portugal. *Environ. Pollut.* **208**, 2–15 (2016). 26239833 10.1016/j.envpol.2015.07.018

[68] Oliveira, M., Slezakova, K., Delerue-Matos, C., Pereira, M. D. C. & Morais, S. Indoor air quality in preschools (3- to 5-year-old children) in the Northeast of Portugal during spring–summer season: pollutants and comfort parameters. *J. Toxicol. Environ. Health A* **80**, 740–755 (2017). 28569620 10.1080/15287394.2017.1286932

[69] Neamtiu, I. A. et al. Assessment of formaldehyde levels in relation to respiratory and allergic symptoms in children from Alba County schools. *Romania. Environ. Monit. Assess.* **191**, 591 (2019). 31446497 10.1007/s10661-019-7768-6

[70] Cabovská, B. et al. Ventilation strategies and indoor air quality in Swedish primary school classrooms. *Build. Environ.* **226**, 109744 (2022).

[71] Wang, J., Smedje, G., Nordquist, T. & Norbäck, D. Personal and demographic factors and change of subjective indoor air quality reported by school children in relation to exposure at Swedish schools: A 2-year longitudinal study. *Sci. Total Environ.* **508**, 288–296 (2015). 25486639 10.1016/j.scitotenv.2014.12.001

[72] U.S. Environmental Protection Agency. Best Practices for Reducing Near-Road Pollution Exposure at Schools (2015). Office of Children’s Health, Washington, D.C., USA https://19january2017snapshot.epa.gov/sites/production/files/2015-10/documents/ochp_2015_near_road_pollution_booklet_v16_508.pdf [last accessed: 12 December 2025]

[73] U.S. Environmental Protection Agency, The Inside Story: A Guide to Indoor Air Quality. (2014) Office of Air and Radiation Office of Radiation and Indoor Air, Washington D.C., USA, https://www.epa.gov/indoor-air-quality-iaq/inside-story-guide-indoor-air-quality [last accessed: 12 December 2025]

[74] Sekar, A., Varghese, G. K. & Ravi Varma, M. K. Analysis of benzene air quality standards, monitoring methods and concentrations in indoor and outdoor environment. *Heliyon* **5**, e02918 (2019). 31844766 10.1016/j.heliyon.2019.e02918 PMC6895577

[75] World Health Organization. Chemical Pollution of Indoor Air and Its Risk for Children’s Health Supplementary Publication to the Screening Tool for Assessment of Health Risks from Combined Exposure to Multiple Chemicals in Indoor Air in Public Settings for Children. (2021), WHO Regional Office for Europe, Copenhagen, Denmark https://iris.who.int/bitstream/handle/10665/341984/9789289055628-eng.pdf?sequence=1 [last accessed: 12 December 2025)

[76] Liu, C., Huang, X. & Li, J. Outdoor benzene highly impacts indoor concentrations globally. *Sci. Total Environ.* **720**, 137640 (2020). 32146409 10.1016/j.scitotenv.2020.137640

[77] Branco, P. T. B. S. et al. A review of relevant parameters for assessing indoor air quality in educational facilities. *Environ. Res.* **261**, 119713 (2024). 39094896 10.1016/j.envres.2024.119713

[78] Salthammer, T., Mentese, S. & Marutzky, R. Formaldehyde in the indoor environment. *Chem. Rev.* **110**, 2536–2572 (2010). 20067232 10.1021/cr800399g PMC2855181

[79] Maximoff, S. N., Mittal, R., Kaushik, A. & Dhau, J. S. Performance evaluation of activated carbon sorbents for indoor air purification during normal and wildfire events. *Chemosphere* **304**, 135314 (2022). 35709843 10.1016/j.chemosphere.2022.135314

[80] Settimo, G. et al. Indoor air quality levels in schools: Role of student activities and no activities. *IJERPH* **17**, 6695 (2020). 32938001 10.3390/ijerph17186695 PMC7559628

[81] Law, C. K., Lai, J. H. K., Ma, X. D. & Sze-To, G. N. Enhancing indoor air quality: Examination of formaldehyde adsorption efficiency of portable air cleaner fitted with chemically-treated activated carbon filters. *Build. Environ.* **263**, 111823 (2024).

[82] Kang, Y.-J. et al. A brief review of formaldehyde removal through activated carbon adsorption. *Appl. Sci.* **12**, 5025 (2022).

[83] World Health Organization, Regional Office for Europe. Methods for sampling and analysis of chemical pollutants in indoor air. (2020). WHO Regional Office for Europe, Copenhagen, Denmark https://iris.who.int/bitstream/handle/10665/334389/9789289055239-eng.pdf [last accessed: 12 December 2025]

