# Supplementary material for: Assessment of health risks from exposure to indoor volatile organic compounds in European educational buildings
Source: Sci Rep. 2026 Jan 28;16:6554. doi: 10.1038/s41598-026-37072-2 (PMC12909993; doi:10.1038/s41598-026-37072-2)
Supplement: Supplementary file 1 — Supplementary Material 1 [file 41598_2026_37072_MOESM1_ESM.docx]

**Assessment of health risks from exposure to indoor volatile organic compounds in European educational buildings**

Anoushka Chatterjee^a*^, László Pál^a*^, Szabolcs Lovas^a^, Martin McKee^b^, Judit Diószegi^a^, Nóra Kovács^a^, Sándor Szűcs^a^

^a^Department of Public Health and Epidemiology, Faculty of Medicine, University of Debrecen, Debrecen, Hungary; ^b^Department of Health Services Research and Policy, London School of Hygiene and Tropical Medicine, London, United Kingdom

*These authors are equal contributors.

E-mail addresses: anoushkachatt@gmail.com (A. Chatterjee), [pal.laszlo@med.unideb.hu](mailto:pal.laszlo@med.unideb.hu) (L. Pál), [lovas.szabolcs@med.unideb.hu](mailto:lovas.szabolcs@med.unideb.hu) (Sz. Lovas), [martin.mckee@lshtm.ac.uk](mailto:martin.mckee@lshtm.ac.uk) (M. McKee), [dioszegi.judit@med.unideb.hu](mailto:dioszegi.judit@med.unideb.hu) (J. Diószegi) [kovacs.nora@med.unideb.hu](mailto:kovacs.nora@med.unideb.hu) (N. Kovács), [szucs.sandor@med.unideb.hu](mailto:szucs.sandor@med.unideb.hu) (S. Szűcs)

*Please address all correspondence to: Dr. László Pál, Department of Public Health and Epidemiology, Faculty of Medicine, University of Debrecen, H–4012 Debrecen, P.O. Box 9, Hungary. (T) 36 52 512 765 (F) 36 52 417 267 (E) [pal.laszlo@med.unideb.hu](mailto:pal.laszlo@med.unideb.hu)

**Supplementary table 1: PODI_adj_ values for respiratory effects due to indoor acetaldehyde exposure in educational buildings in the European countries studied**

| **acetaldehyde exposure** | | | | | | | |
| --- | --- | --- | --- | --- | --- | --- | --- |
| **author** | **country** | **number of buildings studied** | **mean concentration [mg/m^3^]** | **PODI_adj_ respiratory effects** | ***PODI_adj_ cardiovascular effects** | ***PODI_adj_ neurological effects** | ***PODI_adj_ irritation effects** |
| Geiss et al., 2011 | Cyprus | 3 | 0.00643 | 0.0054 |  |  |  |
| Szabados et al., 2021 | Czechia | 12 | 0.00473 | 0.0039 |  |  |  |
| Geiss et al., 2011 | Finland | 3 | 0.00583 | 0.0049 |  |  |  |
| Canha et al., 2015 | France | 17 | 0.00630 | 0.0052 |  |  |  |
| Trocquet et al., 2021 | France | 11 | 0.01213 | 0.0101 |  |  |  |
| Geiss et al., 2011 | Germany | 3 | 0.01464 | 0.0122 |  |  |  |
| Geiss et al., 2011 | Greece | 6 | 0.00897 | 0.0075 |  |  |  |
| Geiss et al., 2011 | Hungary | 6 | 0.00736 | 0.0061 |  |  |  |
| Szabados et al., 2021 | Hungary | 16 | 0.00531 | 0.0044 |  |  |  |
| Geiss et al., 2011 | Ireland | 2 | 0.00421 | 0.0035 |  |  |  |
| Szabados et al, 2021 | Italy | 12 | 0.00574 | 0.0048 |  |  |  |
| Geiss et al., 2011 | Italy | 3 | 0.00650 | 0.0054 |  |  |  |
| Geiss et al., 2011 | Netherlands | 2 | 0.00700 | 0.0058 |  |  |  |
| Szabados et al, 2021 | Poland | 12 | 0.00447 | 0.0037 |  |  |  |
| Fonseca et al., 2021 | Portugal | 20 | 0.00720 | 0.0060 |  |  |  |
| Szabados et al, 2021 | Slovenia | 12 | 0.00616 | 0.0051 |  |  |  |
| Villanueva et al., 2018 | Spain | 18 | 0.00603 | 0.0050 |  |  |  |
| Ninyá et al., 2022 | Spain | 1 | 0.00320 | 0.0027 |  |  |  |
|  |  |  |  |  |  |  |  |
|  |  |  |  |  |  |  |  |
|  |  |  |  |  |  |  |  |
|  |  |  |  |  |  |  |  |
|  |  |  |  |  |  |  |  |
|  |  |  |  |  |  |  |  |

*As point-of-departure values for cardiovascular, neurological and irritation effects have not been reported, adjusted point-of-departure indices (PODI_adj_) could not be calculated.

**Supplementary table 2: PODI_adj_ values for respiratory, cardiovascular, neurological, and irritation effects due to indoor 1,4-dichlorobenzene exposure in educational buildings in the European countries studied**

| **1,4-dichlorobenzene exposure** | | | | | | | |
| --- | --- | --- | --- | --- | --- | --- | --- |
| **author** | **country** | **number of buildings studied** | **mean concentration [mg/m^3^]** | **PODI_adj_ respiratory effects** | **PODI_adj_ cardiovascular effects** | **PODI_adj_ neurological effects** | **PODI_adj_ irritation effects** |
| Canha et al., 2015 | France | 17 | 0.00180 | 0.0015 | 0.0003 | 0.0003 | 0.0003 |
| Lizana et al., 2020 | Spain | 6 | 0.00007 | 0.0001 | 0.0000 | 0.0000 | 0.0000 |
|  |  |  |  |  |  |  |  |
|  |  |  |  |  |  |  |  |
|  |  |  |  |  |  |  |  |
|  |  |  |  |  |  |  |  |
|  |  |  |  |  |  |  |  |
|  |  |  |  |  |  |  |  |
|  |  |  |  |  |  |  |  |
|  |  |  |  |  |  |  |  |
|  |  |  |  |  |  |  |  |
|  |  |  |  |  |  |  |  |
|  |  |  |  |  |  |  |  |
|  |  |  |  |  |  |  |  |
|  |  |  |  |  |  |  |  |
|  |  |  |  |  |  |  |  |
|  |  |  |  |  |  |  |  |
|  |  |  |  |  |  |  |  |
|  |  |  |  |  |  |  |  |
|  |  |  |  |  |  |  |  |
|  |  |  |  |  |  |  |  |
|  |  |  |  |  |  |  |  |
|  |  |  |  |  |  |  |  |
|  |  |  |  |  |  |  |  |
|  |  |  |  |  |  |  |  |
|  |  |  |  |  |  |  |  |
|  |  |  |  |  |  |  |  |
|  |  |  |  |  |  |  |  |

**Supplementary table 3: PODI_adj_ values for respiratory, cardiovascular, neurological, and irritation effects due to indoor ethylbenzene exposure in educational buildings in the European countries studied**

| **ethylbenzene exposure** | | | | | | | |
| --- | --- | --- | --- | --- | --- | --- | --- |
| **author** | **country** | **number of buildings studied** | **mean concentration [mg/m^3^]** | **PODI_adj_ respiratory effects** | **PODI_adj_ cardiovascular effects** | **PODI_adj_ neurological effects** | **PODI_adj_ irritation effects** |
| Geiss et al., 2011 | Cyprus | 3 | 0.00193 | 0.0009 | 0.0001 | 0.0013 | 0.0009 |
| Szabados et al., 2021 | Czechia | 12 | 0.00191 | 0.0009 | 0.0001 | 0.0013 | 0.0009 |
| Geiss et al., 2011 | Finland | 3 | 0.00067 | 0.0003 | 0.0000 | 0.0005 | 0.0003 |
| Canha et al., 2015 | France | 17 | 0.00220 | 0.001 | 0.0001 | 0.0015 | 0.001 |
| Geiss et al., 2011 | Germany | 3 | 0.00126 | 0.0006 | 0.0000 | 0.0009 | 0.0006 |
| Geiss et al., 2011 | Greece | 6 | 0.00456 | 0.0021 | 0.0001 | 0.0031 | 0.0021 |
| Geiss et al., 2011 | Hungary | 6 | 0.00107 | 0.0005 | 0.0000 | 0.0007 | 0.0005 |
| Szabados et al., 2021 | Hungary | 15 | 0.00067 | 0.0003 | 0.0000 | 0.0005 | 0.0003 |
| Geiss et al., 2011 | Ireland | 2 | 0.00061 | 0.0003 | 0.0000 | 0.0004 | 0.0003 |
| Geiss et al., 2011 | Italy | 3 | 0.00590 | 0.0027 | 0.0002 | 0.0041 | 0.0027 |
| Lelpo et al., 2021 | Italy | 1 | 0.00008 | 0.0000 | 0.0000 | 0.0001 | 0.0000 |
| Lucialli et al.,2020 | Italy | 8 | 0.00168 | 0.0008 | 0.0001 | 0.0012 | 0.0008 |
| Marzocca et al., 2017 | Italy | 1 | 0.00076 | 0.0004 | 0.0000 | 0.0005 | 0.0004 |
| Romagnoli et al., 2015 | Italy | 1 | 0.00054 | 0.0003 | 0.0000 | 0.0004 | 0.0003 |
| Szabados et al, 2021 | Italy | 11 | 0.00378 | 0.0018 | 0.0001 | 0.0026 | 0.0018 |
| Geiss et al., 2011 | Netherlands | 2 | 0.00098 | 0.0005 | 0.0000 | 0.0007 | 0.0005 |
| Szabados et al, 2021 | Poland | 11 | 0.00138 | 0.0006 | 0.0000 | 0.0010 | 0.0006 |
| Fonseca et al., 2021 | Portugal | 20 | 0.00390 | 0.0018 | 0.0001 | 0.0027 | 0.0018 |
| Pegas et al., 2011 | Portugal | 14 | 0.00252 | 0.0012 | 0.0001 | 0.0017 | 0.0012 |
| Kovacs et al., 2013 | Romania | 5 | 0.01672 | 0.0078 | 0.0005 | 0.0115 | 0.0078 |
| Szabados et al, 2021 | Slovenia | 11 | 0.00067 | 0.0003 | 0.0000 | 0.0005 | 0.0003 |
| Lizana et al., 2020 | Spain | 6 | 0.00078 | 0.0004 | 0.0000 | 0.0005 | 0.0004 |
| Ninyá et al., 2022 | Spain | 1 | 0.00247 | 0.0011 | 0.0001 | 0.0017 | 0.0011 |
| Vallecillos et al., 2020 | Spain | 1 | 0.00040 | 0.0002 | 0.0000 | 0.0003 | 0.0002 |
| Villanueva et al., 2018 | Spain | 18 | 0.00084 | 0.0004 | 0.0000 | 0.0006 | 0.0004 |
|  |  |  |  |  |  |  |  |
|  |  |  |  |  |  |  |  |
|  |  |  |  |  |  |  |  |

**Supplementary table 4: PODI_adj_ values for respiratory, cardiovascular, neurological, and irritation effects due to indoor o, m, p-xylene exposure in educational buildings in the European countries studied**

| **o, m, p-xylene exposure** | | | | | | | |
| --- | --- | --- | --- | --- | --- | --- | --- |
| **author** | **country** | **number of buildings studied** | **mean concentration [mg/m^3^]** | **PODI_adj_ respiratory effects** | **PODI_adj_ cardiovascular effects** | **PODI_adj_ neurological effects** | **PODI_adj_ irritation effects** |
| Geiss et al., 2011 | Cyprus | 3 | 0.00512 | 0.0035 | 0.0042 | 0.0035 | 0.0035 |
| Szabados et al., 2021 | Czechia | 12 | 0.00525 | 0.0026 | 0.0032 | 0.0026 | 0.0026 |
| Geiss et al., 2011 | Finland | 3 | 0.00147 | 0.0013 | 0.0015 | 0.0013 | 0.0013 |
| Canha et al., 2015 | France | 17 | 0.00440 | 0.0030 | 0.0036 | 0.0030 | 0.0030 |
| Geiss et al., 2011 | Germany | 3 | 0.00242 | 0.0017 | 0.0020 | 0.0017 | 0.0017 |
| Geiss et al., 2011 | Greece | 6 | 0.01133 | 0.0072 | 0.0088 | 0.0072 | 0.0072 |
| Geiss et al., 2011 | Hungary | 6 | 0.00277 | 0.0024 | 0.0030 | 0.0024 | 0.0024 |
| Szabados et al., 2021 | Hungary | 15 | 0.00150 | 0.0007 | 0.0009 | 0.0007 | 0.0007 |
| Geiss et al., 2011 | Ireland | 2 | 0.00209 | 0.0016 | 0.0019 | 0.0016 | 0.0016 |
| Lelpo et al., 2021 | Italy | 1 | 0.00018 | 0.0001 | 0.0001 | 0.0001 | 0.0001 |
| Marzocca et al., 2017 | Italy | 1 | 0.00255 | 0.0013 | 0.0015 | 0.0013 | 0.0013 |
| Romagnoli et al., 2015 | Italy | 1 | 0.00353 | 0.0017 | 0.0021 | 0.0017 | 0.0017 |
| Szabados et al, 2021 | Italy | 11 | 0.01515 | 0.0075 | 0.0091 | 0.0075 | 0.0075 |
| Geiss et al., 2011 | Netherlands | 2 | 0.00258 | 0.0013 | 0.0016 | 0.0013 | 0.0013 |
| Szabados et al, 2021 | Poland | 11 | 0.00438 | 0.0022 | 0.0026 | 0.0022 | 0.0022 |
| Kovacs et al., 2013 | Romania | 5 | 0.00994 | 0.0049 | 0.0060 | 0.0049 | 0.0049 |
| Szabados et al, 2021 | Slovenia | 11 | 0.00146 | 0.0007 | 0.0009 | 0.0007 | 0.0007 |
| Geiss et al., 2011 | Italy | 3 | 0.02410 | 0.0119 | 0.0145 | 0.0119 | 0.0119 |
| Fonseca et al., 2021 | Portugal | 20 | 0.01600 | 0.0079 | 0.0096 | 0.0079 | 0.0079 |
| Pegas et al., 2011 | Portugal | 14 | 0.01287 | 0.0063 | 0.0078 | 0.0063 | 0.0063 |
| Lizana et al., 2020 | Spain | 6 | 0.00306 | 0.0015 | 0.0018 | 0.0015 | 0.0015 |
| Ninyá et al., 2022 | Spain | 1 | 0.00735 | 0.0036 | 0.0044 | 0.0036 | 0.0036 |
| Vallecillos et al., 2020 | Spain | 1 | 0.00127 | 0.0006 | 0.0008 | 0.0006 | 0.0006 |
|  |  |  |  |  |  |  |  |
|  |  |  |  |  |  |  |  |
|  |  |  |  |  |  |  |  |
|  |  |  |  |  |  |  |  |
|  |  |  |  |  |  |  |  |

**Supplementary table 5: PODI_adj_ values for respiratory, cardiovascular, neurological, and irritation effects due to indoor styrene exposure in educational buildings in the European countries studied**

| **styrene exposure** | | | | | | | |
| --- | --- | --- | --- | --- | --- | --- | --- |
| **author** | **country** | **number of buildings studied** | **mean concentration [mg/m^3^]** | **PODI_adj_ respiratory effects** | **PODI_adj_ cardiovascular effects** | **PODI_adj_ neurological effects** | **PODI_adj_ irritation effects** |
| Geiss et al., 2011 | Cyprus | 3 | 0.00015 | 0.0005 | 0.0000 | 0.0001 | 0.0001 |
| Szabados et al., 2021 | Czechia | 12 | 0.00052 | 0.0018 | 0.0001 | 0.0002 | 0.0004 |
| Geiss et al., 2011 | Finland | 3 | 0.00015 | 0.0005 | 0.0000 | 0.0001 | 0.0001 |
| Canha et al., 2015 | France | 17 | 0.00150 | 0.0053 | 0.0002 | 0.0005 | 0.0011 |
| Geiss et al., 2011 | Germany | 3 | 0.00036 | 0.0013 | 0.0001 | 0.0001 | 0.0003 |
| Geiss et al., 2011 | Greece | 6 | 0.00013 | 0.0005 | 0.0000 | 0.0000 | 0.0001 |
| Geiss et al., 2011 | Hungary | 6 | 0.00015 | 0.0005 | 0.0000 | 0.0001 | 0.0001 |
| Geiss et al., 2011 | Ireland | 2 | 0.00015 | 0.0005 | 0.0000 | 0.0001 | 0.0001 |
| Marzocca et al., 2017 | Italy | 1 | 0.00016 | 0.0006 | 0.0000 | 0.0001 | 0.0001 |
| Szabados et al, 2021 | Italy | 11 | 0.00070 | 0.0025 | 0.0001 | 0.0002 | 0.0005 |
| Geiss et al., 2011 | Netherlands | 2 | 0.00020 | 0.0007 | 0.0000 | 0.0001 | 0.0001 |
| Szabados et al, 2021 | Poland | 11 | 0.00046 | 0.0016 | 0.0001 | 0.0002 | 0.0003 |
| Fonseca et al., 2021 | Portugal | 20 | 0.00100 | 0.0035 | 0.0001 | 0.0004 | 0.0007 |
| Kovacs et al., 2013 | Romania | 5 | 0.00866 | 0.0305 | 0.0013 | 0.0030 | 0.0061 |
| Szabados et al, 2021 | Slovenia | 11 | 0.00031 | 0.0011 | 0.0000 | 0.0001 | 0.0002 |
| Lizana et al., 2020 | Spain | 6 | 0.00095 | 0.0033 | 0.0001 | 0.0003 | 0.0007 |
| Ninyá et al., 2022 | Spain | 1 | 0.00085 | 0.0030 | 0.0001 | 0.0003 | 0.0006 |
| Vallecillos et al., 2020 | Spain | 1 | 0.00036 | 0.0013 | 0.0001 | 0.0001 | 0.0003 |
| Villanueva et al., 2018 | Spain | 18 | 0.00430 | 0.0151 | 0.0006 | 0.0015 | 0.0030 |
|  |  |  |  |  |  |  |  |
|  |  |  |  |  |  |  |  |
|  |  |  |  |  |  |  |  |
|  |  |  |  |  |  |  |  |
|  |  |  |  |  |  |  |  |
|  |  |  |  |  |  |  |  |
|  |  |  |  |  |  |  |  |
|  |  |  |  |  |  |  |  |
|  |  |  |  |  |  |  |  |

**Supplementary table 6: PODI_adj_ values for respiratory, cardiovascular, neurological, and irritation effects due to indoor toluene exposure in educational buildings in the European countries studied**

| **toluene exposure** | | | | | | | |
| --- | --- | --- | --- | --- | --- | --- | --- |
| **author** | **country** | **number of buildings studied** | **mean concentration [mg/m^3^]** | **PODI_adj_ respiratory effects** | **PODI_adj_ cardiovascular effects** | **PODI_adj_ neurological effects** | **PODI_adj_ irritation effects** |
| Geiss et al., 2011 | Cyprus | 3 | 0.01818 | 0.0002 | 0.0001 | 0.0001 | 0.0001 |
| Szabados et al., 2021 | Czechia | 12 | 0.00933 | 0.0012 | 0.001 | 0.0006 | 0.0008 |
| Geiss et al., 2011 | Finland | 3 | 0.00505 | 0.0007 | 0.0006 | 0.0003 | 0.0004 |
| Canha et al., 2015 | France | 17 | 0.00520 | 0.0007 | 0.0006 | 0.0003 | 0.0005 |
| Geiss et al., 2011 | Germany | 3 | 0.00574 | 0.0008 | 0.0006 | 0.0004 | 0.0005 |
| Geiss et al., 2011 | Greece | 5 | 0.02558 | 0.0034 | 0.0028 | 0.0016 | 0.0023 |
| Geiss et al., 2011 | Hungary | 6 | 0.00669 | 0.0009 | 0.0007 | 0.0004 | 0.0006 |
| Szabados et al., 2021 | Hungary | 15 | 0.00332 | 0.0004 | 0.0004 | 0.0002 | 0.0003 |
| Geiss et al., 2011 | Ireland | 2 | 0.00208 | 0.0003 | 0.0002 | 0.0001 | 0.0002 |
| Geiss et al., 2011 | Italy | 3 | 0.01890 | 0.0025 | 0.0021 | 0.0012 | 0.0017 |
| Lelpo et al., 2021 | Italy | 1 | 0.00081 | 0.0001 | 0.0001 | 0.0000 | 0.0001 |
| Lucialli et al.,2020 | Italy | 8 | 0.00447 | 0.0006 | 0.0005 | 0.0003 | 0.0004 |
| Marzocca et al.et al., 2017 | Italy | 1 | 0.00154 | 0.0002 | 0.0002 | 0.0001 | 0.0001 |
| Romagnoli et al., 2015 | Italy | 1 | 0.00325 | 0.0004 | 0.0004 | 0.0002 | 0.0003 |
| Szabados et al, 2021 | Italy | 11 | 0.03660 | 0.0049 | 0.0040 | 0.0023 | 0.0032 |
| Geiss et al., 2011 | Netherlands | 2 | 0.00300 | 0.0004 | 0.0003 | 0.0002 | 0.0003 |
| Szabados et al, 2021 | Poland | 11 | 0.00781 | 0.0010 | 0.0009 | 0.0005 | 0.0007 |
| Fonseca et al., 2021 | Portugal | 20 | 0.01440 | 0.0019 | 0.0016 | 0.0009 | 0.0013 |
| Pegas et al., 2011 | Portugal | 14 | 0.00460 | 0.0006 | 0.0005 | 0.0003 | 0.0004 |
| Pegas et al., 2012 | Portugal | 2 | 0.00277 | 0.0004 | 0.0003 | 0.0002 | 0.0002 |
| Kovacs et al., 2013 | Romania | 5 | 0.02363 | 0.0031 | 0.0026 | 0.0015 | 0.0021 |
| Szabados et al, 2021 | Slovenia | 11 | 0.00651 | 0.0009 | 0.0007 | 0.0004 | 0.0006 |
| Lizana et al., 2020 | Spain | 6 | 0.00581 | 0.0008 | 0.0006 | 0.0004 | 0.0005 |
| Ninyá et al., 2022 | Spain | 1 | 0.01195 | 0.0016 | 0.0013 | 0.0007 | 0.0011 |
| Vallecillos et al., 2020 | Spain | 1 | 0.00474 | 0.0006 | 0.0005 | 0.0003 | 0.0004 |
| Villanueva et al., 2018 | Spain | 18 | 0.00396 | 0.0005 | 0.0004 | 0.0002 | 0.0004 |
|  |  |  |  |  |  |  |  |

**Supplementary table 7: PODI_adj_ values for respiratory, cardiovascular, neurological, and irritation effects due to indoor trichloroethylene exposure in educational buildings in the European countries studied**

| **trichloroethylene exposure** | | | | | | | |
| --- | --- | --- | --- | --- | --- | --- | --- |
| **author** | **country** | **number of buildings studied** | **mean concentration [mg/m^3^]** | **PODI_adj_ respiratory effects** | **PODI_adj_ cardiovascular effects** | **PODI_adj_ neurological effects** | **PODI_adj_ irritation effects** |
| Canha et al., 2015 | France | 17 | 0.00230 | 0.0007 | 0.0001 | 0.0051 | 0.0007 |
| Kovacs et al., 2013 | Romania | 5 | 0.00546 | 0.0016 | 0.0002 | 0.0122 | 0.0016 |
| Lizana et al., 2020 | Spain | 6 | 0.00006 | 0.0000 | 0.0000 | 0.0001 | 0.0000 |
|  |  |  |  |  |  |  |  |
|  |  |  |  |  |  |  |  |
|  |  |  |  |  |  |  |  |
|  |  |  |  |  |  |  |  |
|  |  |  |  |  |  |  |  |
|  |  |  |  |  |  |  |  |
|  |  |  |  |  |  |  |  |
|  |  |  |  |  |  |  |  |
|  |  |  |  |  |  |  |  |
|  |  |  |  |  |  |  |  |
|  |  |  |  |  |  |  |  |
|  |  |  |  |  |  |  |  |
|  |  |  |  |  |  |  |  |
|  |  |  |  |  |  |  |  |
|  |  |  |  |  |  |  |  |
|  |  |  |  |  |  |  |  |
|  |  |  |  |  |  |  |  |
|  |  |  |  |  |  |  |  |
|  |  |  |  |  |  |  |  |
|  |  |  |  |  |  |  |  |
|  |  |  |  |  |  |  |  |
|  |  |  |  |  |  |  |  |
|  |  |  |  |  |  |  |  |
|  |  |  |  |  |  |  |  |
